# Anorexia Nervosa, Schizophrenia, and Autism Spectrum Disorder: A Review of the Role of Tanycytes in Individual and Social Behavior

**DOI:** 10.7759/cureus.112030

**Published:** 2026-07-04

**Authors:** Prasad Dalvi, Olivia Kelly, Leah Grady, Natalie Carris, Jillian Sullivan, He Liu

**Affiliations:** 1 Biology Department, Gannon University, Erie, USA

**Keywords:** anorexia nervosa (an), autism spectrum disorder (asd), glial cells, schizophrenia (scz), tanycytes

## Abstract

Located along the third ventricle of the hypothalamus, tanycytes are a type of glial cell that are prominent components of metabolic, homeostatic, and immunological signaling and transport. Behavioral deficits observed in disorders such as anorexia nervosa (AN), schizophrenia (SCZ), and autism spectrum disorder (ASD) have been observed and can be linked to the various functions of tanycytes. This review investigates the association between tanycytes and AN, SCZ, and ASD, potentially giving insight into the pathogenesis of these disorders. Specifically, attention is paid to the role of tanycytes in immunological and metabolic pathways, as well as their involvement in the hypothalamic parenchyma via extended processes. Knowledge about the basic molecular and behavioral pathology of AN, SCZ, and ASD may help establish the link between tanycytes and behavior, both on the individual and social level. More mechanistic studies are required, as currently available data are just hypothesis-generating and not mechanistically established.

## Introduction and background

Tanycyte structure and function

**Figure 1:**
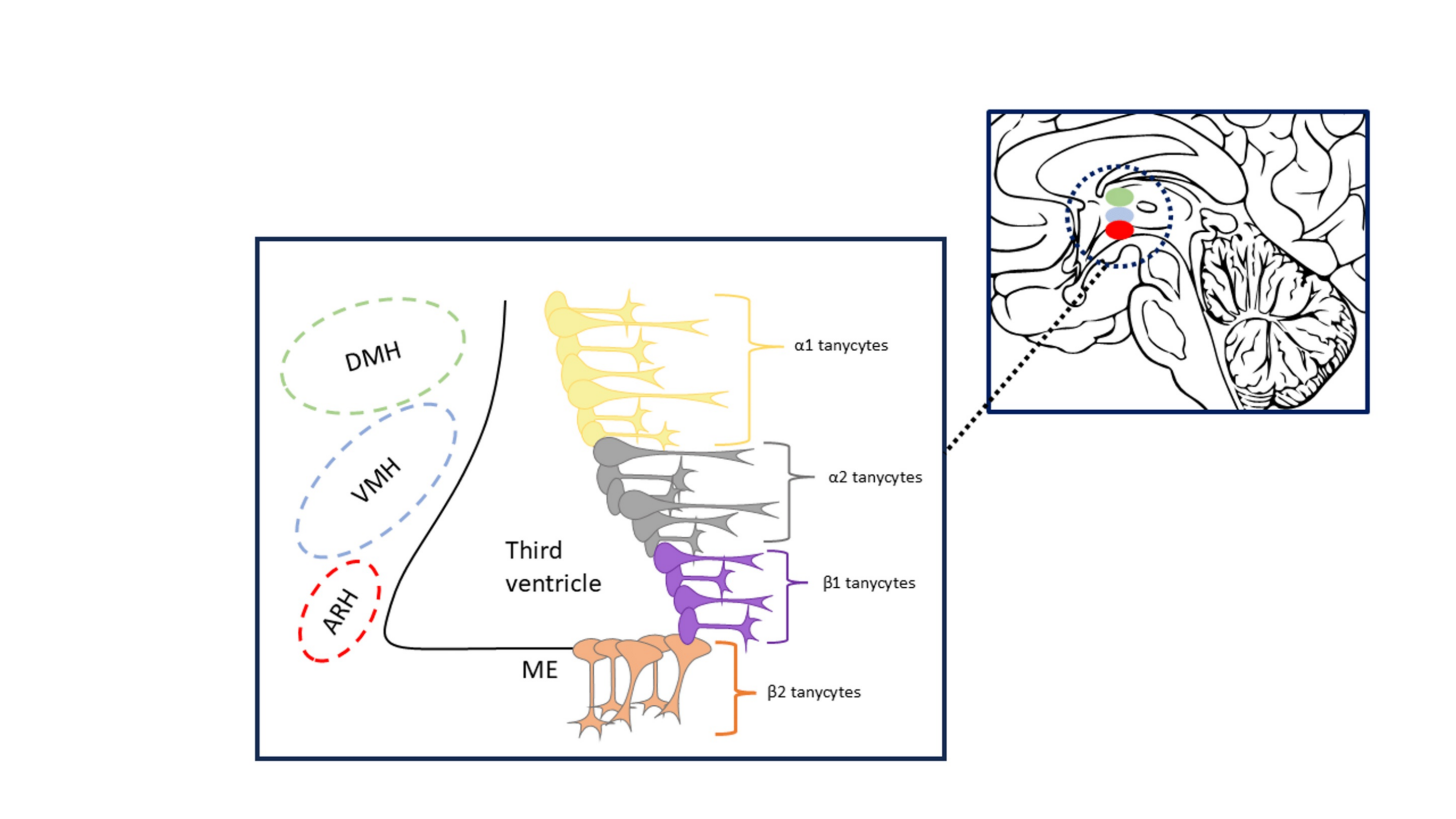
Simplified not-to-scale schematic diagram of tanycyte morphologies and their relative locations in the third ventricle of the hypothalamus Long processes extend from the cell bodies along the third ventricle and insert into the hypothalamic parenchyma. The cell bodies are located in the median eminence (ME), dorsomedial nucleus of the hypothalamus (DMH), ventromedial nucleus of the hypothalamus (VMH), and arcuate nucleus of the hypothalamus (ARH). The organization of these cell bodies creates tight junctions. A schematic representation of various morphological types of tanycytes is shown. Figure/image credit: Leah Grady, Olivia Kelly, and Prasad Dalvi. The figure is prepared using PowerPoint. No AI-assisted tool was used for creating the image.

Various glial cell types, including astrocytes, tanycytes, microglia, and NG2 glia, are located in the hypothalamus. Glial cells play important roles in energy metabolism [1-4]. Tanycytes are distinguished from other glial cells by their long processes, which extend from their cell bodies and insert into the hypothalamic parenchyma (Figure 1). Cell bodies of tanycytes are lined up all along the third ventricle and classified by their locations in the median eminence (ME) of the hypothalamus, the dorsomedial nucleus of the hypothalamus (DMH), the ventromedial nucleus of the hypothalamus (VMH), and in both the ventromedial and dorsomedial areas of the arcuate nucleus of the hypothalamus (ARH) (Figure 1) [5]. This positioning of the tanycytes allows the formation of tight junctions, which prevent the diffusion of blood-borne molecules from highly permeable fenestrated capillaries to the rest of the brain [6,7]. In particular, ME tanycytes and ARH tanycytes that come in contact with fenestrated capillaries can form tight junctions due to their susceptibility to blood-borne molecules [6-8].

Tanycytes in behavioral disorders

Tanycytes are hypothesized to play a role in the development and onset of individual and social behavioral neuropathology. Major disorders such as anorexia nervosa (AN), schizophrenia (SCZ), and autism spectrum disorder (ASD) have been studied in relation to tanycyte morphology and physiology. These conditions were chosen because, despite the limited mechanistic data available, they represent the most consistently discussed neuropsychiatric disorders in the emerging literature, linking hypothalamic regulation, metabolic signaling, and social/behavioral dysfunction. We propose that tanycytes may serve as a convergent interface between metabolic, hormonal, and neural signaling systems relevant to these disorders. Before considering the role of tanycytes, key characteristics of these three disorders must be reviewed.

Largely considered to be associated with societal expectations for women, AN is a leading problem in young adolescents, particularly females. AN generally begins with manifestation in eating patterns and obsessions concerning weight loss; if prolonged, these symptoms can lead to severe health issues and even suicide [9]. Aside from these environmental factors, AN also involves biological and psychological factors in its diagnosis, including possible genetic predispositions toward obsessive and compulsive behaviors, anxiety, and the overall likelihood of developing AN [10].

Generally, social cognitive deficits are a hallmark pathology of both ASD and SCZ, leading to their classification as neurodevelopmental disorders. However, a key difference between ASD and SCZ is their respective ages of onset. ASD generally begins with a display of language delays in early childhood, during the second to fourth years of age, and SCZ first makes a clinical appearance in late adolescence [11]. According to the Diagnostic and Statistical Manual of Mental Disorders, Fifth Edition (DSM-5), ASD has hallmark characteristics of restrictive and repetitive patterns of behavior [9]. Several studies have consistently observed a pattern of drastically overgrown brain volume during both infancy and early childhood [12,13]. Children with ASD also display accelerated brain development [14]. From a neurobiological standpoint, the cognitive symptoms of SCZ are hypothesized to result from the low activity of the N-methyl-D-aspartate receptor on gamma-aminobutyric acid inhibitory interneurons in the prefrontal cortex. Psychosis is the most observed sign of SCZ, frequently appearing between ages 18 and 25 years, a period when the prefrontal cortex is still developing. Interestingly, the genetic abnormalities associated with SCZ bear similarities to those found in ASD and other neurodevelopmental disorders [15]. SCZ affects both excitatory and inhibitory neurons in the central nervous system, making neurons the primary source of pathology. Consequently, SCZ may not be restricted to a specific brain structure [16]. Tanycytes provide both structure and protection for the hypothalamus - a major regulatory center in the brain. The close proximity of tanycytes to the hypothalamus enables them to intercept and regulate glucose sensing and signaling. This narrative review will discuss and review the possible etiological contributions of tanycytes to major neuropsychiatric disorders such as AN, SCZ, and ASD. We have included up-to-date available comprehensive literature in this article. As such, the role of tanycytes in neuropsychiatric disorders is a new topic, and not much research has been conducted. More mechanistic studies are required, as currently available data are just hypothesis-generating and not mechanistically established.

## Review

Anorexia nervosa

*Anorexia Nervosa*​​​​​​​* Pathophysiology*

AN is a psychological disorder characterized by reduced eating behaviors, significant weight loss, and diminished self-esteem. Common causes of eating disorders include biological causes, such as serotonin dysfunction, and psychological causes, such as intense body dissatisfaction, obsessive personality traits, experiences of abuse, and cultural influences [10]. Several studies have monitored the immune response (marked by cytokines, which drive inflammation) in patients with AN. The levels of tumor necrosis factor-alpha (TNF-α) and interleukin-6 (IL-6) have been found to be elevated in some patients with AN compared with control groups across multiple experiments [17-20]. It is, however, important to note that decreased levels of TNF-α and IL-6 were also found in patients with AN compared with control groups [21]. Interestingly, these two pro-inflammatory cytokines have long been associated with energy balance and metabolism [22]. The presence of IL-6 in the hypothalamus has recently been highlighted as a necessity in reducing food intake, specifically in males [23]. The same study found that about half of the IL-6 was produced by neurons, and the other half by astrocytes and microglia. There was IL-6 receptor activity in the VMH, suggesting that cells (possibly tanycytes) in this area might be activated by the increased release of IL-6. This process may trigger some mechanism that reduces food intake, given the established role of tanycytes in appetite regulation [23].

The tumor necrosis factor (TNF) cytokine family as a whole has been widely researched, and its presence in the brain has been associated with decreased insulin sensitivity and appetite, further validating the previously mentioned findings [24]. Upregulated IL-6 and TNF-α in the brain may influence nearby tanycytes to regulate the food intake machinery, such as regulation of leptin and ghrelin, glucose transport, and taste receptor activation, resulting in a subdued hunger response in patients with AN. Upregulation of IL-6 and TNF-α cytokines may be due to the presence of concurrent infection in patients with AN; this upregulation might also suggest AN predisposition based on autoimmune-related mechanisms. A recent review article supports these hypotheses and suggests a potential comorbidity between AN and autoimmune disorders [25]. Further investigation is recommended into acute immune dysfunction, increasing the likelihood of psychological disorder development [25].

Inflammation-Induced Anorexia Nervosa

Anorexia, apart from being present in psychological disorder AN, can also refer to inflammation-induced anorexia, a common condition where an individual loses appetite following infection. Inflammation triggered by infection involves the recruitment of immune cells through immunological synapsing and cytokine signaling. Cytokines such as interferons (IFNs), TNFs, and interleukins (ILs) all contribute to inflammation observed in inflammation-induced anorexia. IFNs drive the production of anti-viral gene products, while TNFs mediate cell proliferation and death. ILs drive cell activation, differentiation, and gene expression. Specifically, IL-1B promotes pro-inflammatory signals, including interferon regulatory factor 3 (IRF3), interferon regulatory factor 7 (IRF7), nuclear factor kappa B (NF-kB), and activator protein 1 (AP-1). These transcription factors drive increased expression of certain genes or can cause other cells to release cytokines [26]. Induction of *PTGS2* gene expression by IL-1B and NF-kB signaling in mice is responsible for inflammation-induced anorexia; this signaling results in the release of anorexigenic prostaglandin E2 from tanycytes [27]. Because tanycytes are responsive to IL-1B signaling and can induce anorexia, tanycyte involvement in AN is possible, and more investigation is warranted [27].

Tanycytes and Anorexia Nervosa​​​​​​​: Appetite Regulation

In addition to their protective role in the brain, ME tanycytes near the ARH intercept and transport endocrine signaling of the major food-intake-related hormones leptin and ghrelin [28-30]. Studies were conducted to highlight the pathway of leptin signaling (which is involved in regulating hunger); when administered, leptin moves from the bloodstream into fenestrated capillaries, where it is taken up by ME tanycytes. Leptin can then diffuse through the non-ciliated ME and ARH tanycytic cell bodies, ultimately reaching the ARH and perpetuating anorexigenic signaling [30]. Ghrelin, the hormone responsible for inducing hunger, is also taken up in the ARH area; studies support that, besides the ARH neurons, ARH tanycytes may also play a role in the ghrelin pathway [29,31,32]. Ghrelin and leptin secretions work in opposition to form hunger and satiation signals in the body. A study monitoring leptin levels in patients with AN found that leptin increased significantly following weight gain, illustrating the role of leptin in hunger regulation [33]. Another study measured ghrelin levels in patients with AN during fasting and insulin infusion; ghrelin levels decreased after insulin treatment, indicating ghrelin’s involvement in the body’s hunger response [34]. The role of these hormones in AN [28-34] and the release of anorexigenic prostaglandin E2 from tanycytes in appetite regulation suggest that tanycytes might also be involved in AN [27].

Tanycytes are located in the VMH, the region of the hypothalamus responsible for hunger and satiation. Recently, two studies were conducted to determine the exact role of tanycytes in metabolism. In the presence of glucose, Ca^2+^ in the hypothalamus was greatly increased, and tanycytes seem to be the likely cause of this increase. Tanycytes were found to express signal transducers, such as glucose transporter 2 (GLUT2) and glucokinase, which would allow for the transport and recognition of glucose in the hypothalamus. These tanycytes also exhibited increased Ca^2+^ [35]. Another mechanism that would exhibit increased Ca^2+^ in tanycytes involves the glucose-induced activation of taste receptors, such as Tas1r2 and Tas1r3, which are thought to be present in tanycytes [36]. In addition to the expression of GLUT2 and other signal transducers, monocarboxylate transporters (MCTs) 1 and 4 were also found to be highly expressed in certain tanycytes [37]. Expression of these MCTs is thought to be localized in ARH tanycytes for efficient transport of lactate to produce the anti-hunger response due to their close proximity to glucose-excited neurons [37]. Tanycytes likely regulate the hunger response through their efficient transport of glucose and lactate. Beyond their contribution to metabolism, these cells may also be key to understanding various eating patterns and appetite regulation mechanisms. However, showing that tanycytes may participate in appetite regulation or inflammation-induced anorexia does not by itself establish a meaningful role in the core psychopathology of AN, and more research is warranted.

*Tanycytes and Anorexia Nervosa*​​​​​​​*: The Blood-Brain Barrier and the Blood-Cerebrospinal Fluid Barrier*

The brain is surrounded by two barrier systems consisting of tight junctions: the blood-brain barrier (BBB) and the blood-cerebrospinal fluid barrier (BCSFB). While both the BBB and BCSFB prevent the diffusion of water-soluble molecules between cells, they serve distinct functions. The BBB functions to separate the blood and the brain, whereas the BCSFB separates the blood from the cerebrospinal fluid (CSF). Some other components of the BCSFB, specifically around the third ventricle, are possibly tanycytes or tanycyte-like cells [38]. A study confirmed the presence of ependymal cells in the ME with characteristic long processes that resemble tanycytes, and these tanycyte-like cells are proposed to be involved in macromolecule transport between the various compartments of the brain [7]. Tanycytes, specifically those in the ARH, VMH, and DMH, are also associated with the more restrictive BBB, where they contact fenestrated vessels with their long processes [6]. Both the BBB and BCSFB are integral parts of the immunological protection of the brain. It is possible that tanycytes play a role in intersecting various immune responses and appetite regulation signaling.

Tanycytes and autism spectrum disorder

*Connections Between Autism Spectrum Disorder* ​​​​​​​*and the Neuroendocrine System*

**Figure 2:**
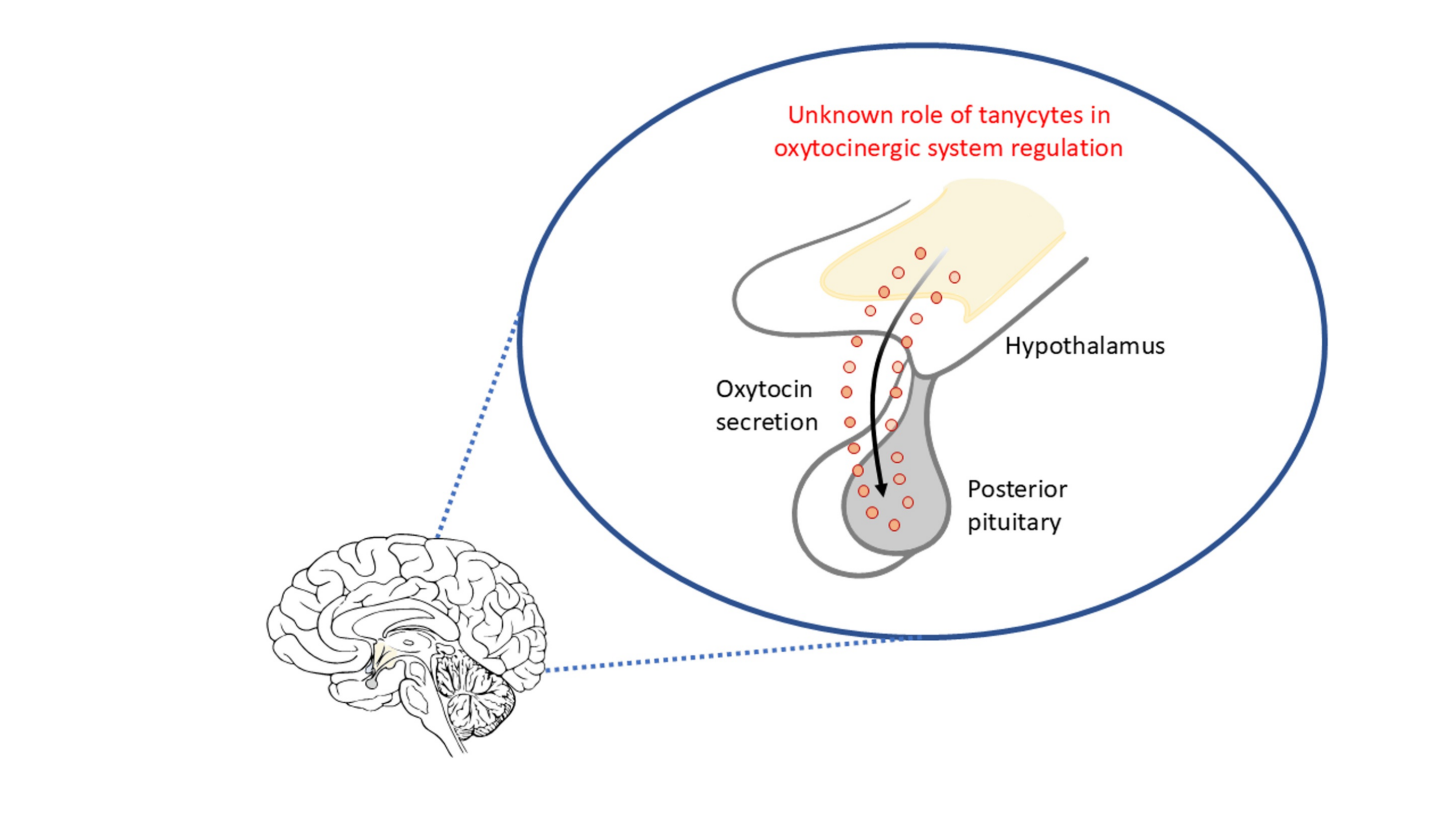
Simplified not-to-scale schematic diagram of the oxytocinergic system in a normal brain Hypothalamic oxytocinergic neurons synthesize and release oxytocin to the posterior pituitary gland for further release into systemic circulation. The role of tanycytes in oxytocinergic system regulation needs to be investigated. Figure/image credit: Leah Grady, Olivia Kelly, and Prasad Dalvi. The figure is prepared using PowerPoint. No AI-assisted tool was used for creating the image.

**Figure 3:**
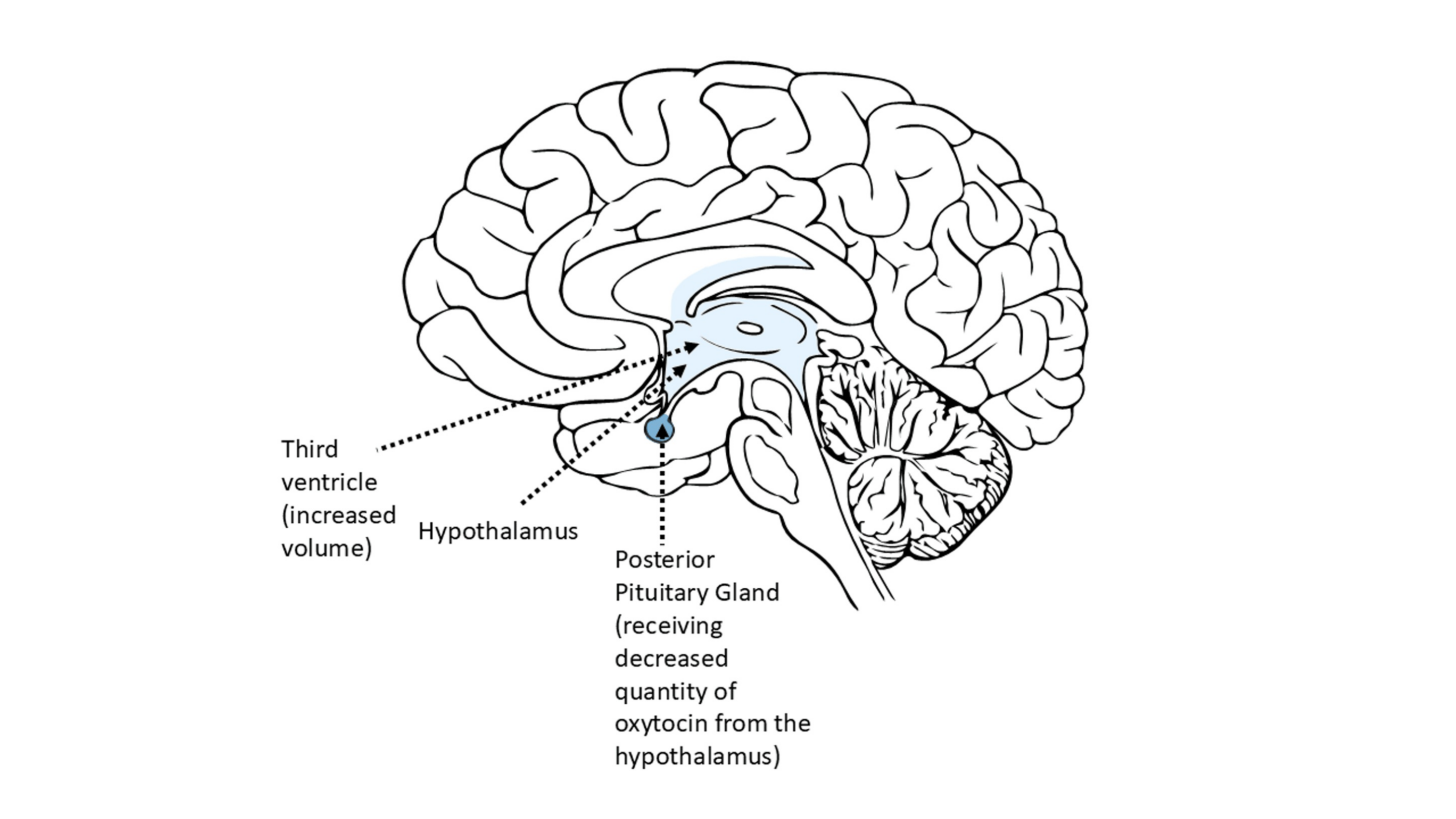
Simplified not-to-scale illustrative schematic of reported hypothalamic/third-ventricle changes associated with autism spectrum disorder in selected imaging studies Shown is decreased oxytocin release from the hypothalamus to the posterior pituitary gland and increased volume of the third ventricle. It is hypothesized that tanycytes may play a role in this abnormality. More research is warranted. Figure/image credit: Leah Grady, Olivia Kelly, and Prasad Dalvi. The figure is prepared using PowerPoint. No AI-assisted tool was used for creating the image.

Knowledge about the basic molecular and behavioral pathology of ASD allows further investigation into the role of tanycytes in its pathogenesis. Tanycytes are hypothesized to act as a complex part of the neuroendocrine system, influencing activity in the third ventricle of the hypothalamus. Research exploring the link to neuropsychiatric disorders has identified the association between dysfunction in the oxytocinergic system and the social deficits observed in ASD [39]. In this context, hypothalamic synthesis and release of oxytocin to the posterior pituitary may be involved (Figure 2). Brain scans of patients with ASD show decreased hypothalamic gray matter density and a significant increase in third ventricle volume, alongside hypothalamic atrophy that leads to decreased synthesis and release of oxytocin from the hypothalamus to the posterior pituitary gland (Figure 3). These observations of decreased hypothalamic gray matter, and therefore most likely cell mass, suggest potential involvement of neuronal and glial cells, including tanycytes, due to their high metabolic and neuroendocrine activity [40]. More research is needed to test the hypothesis that tanycytes regulate the oxytocinergic system.

*Involvement of Tanycytes in Autism Spectrum Disorder*​​​​​​​*​​*​​​​​*: Altered Synaptic Pruning and Metal Homeostasis*

In addition to oxytocinergic system dysfunction, it is hypothesized that tanycytes may contribute to the disruption of normal synaptic pruning in individuals with ASD. C1q is a protein complex of the classical pathway of complement activation, and elevated levels of this complement protein are likely promotors of synaptic pruning. Aside from this association, C1q peripheral serum levels have been found to be increased in young patients with ASD [41]. Moreover, CTRP9, a family of C1q, is associated with the regulation of hypothalamic expression of the orexigenic peptides neuropeptide Y and agouti-related protein [42]. Interactions between the complement system and the hypothalamus point to the involvement of the BBB and BSCFB, which have already been established as major areas of tanycyte signaling. Whether tanycytes synthesize or regulate complement system via C1q protein complex needs to be further investigated.

The expression of CuZn superoxide dismutase 1 in tanycytes enables these cells to regulate metal homeostasis [43]. Imbalances in zinc and copper have been associated with decreased function of *FOXP2*, a gene responsible for some speech and language disorders and a possible cause of speech and language delays observed in patients with ASD [44]. Interestingly, patients with ASD not only experience difficulties with speech and language but also tend to exhibit low zinc and copper levels in their hair and blood [45-47].

**Figure 4:**
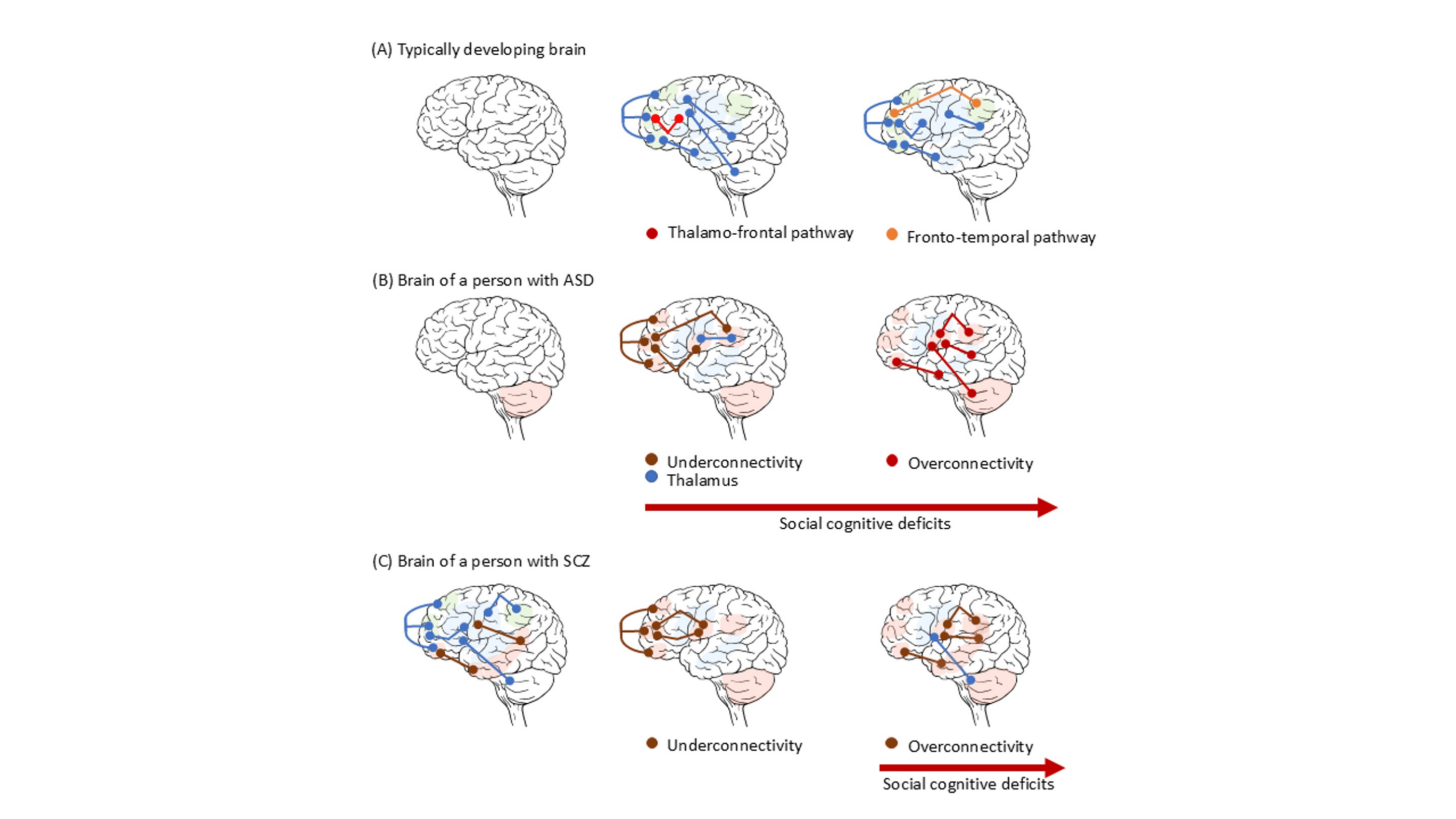
Simplified not-to-scale schematic conceptual model of brain connectivity in the cerebellum and prefrontal cortex (A) In the typically developing brain, normal thalamo-frontal and fronto-temporal pathways are present. (B) In the brain of a patient with autism spectrum disorder, both underconnectivity and overconnectivity may contribute to social cognitive deficits. (C) In the brain of a patient with schizophrenia, overconnectivity may contribute to social cognitive deficits. Figure/image credit: Leah Grady, Olivia Kelly, and Prasad Dalvi. The figure is prepared using PowerPoint. No AI-assisted tool was used for creating the image.

Tanycytes are believed to be involved in certain aspects of ASD. Children diagnosed with ASD between the ages of two and four years often show a significant increase in the volume of their cerebellum [48]. Furthermore, patients with ASD commonly display impaired cellular metal homeostasis, which leads to hyperactivation in the cerebellum (Figure 4). This neuronal hyperactivation can then cause M1 polarization of microglia, resulting in neuroinflammation and leading to dysfunction in the body [49]. Such neuroinflammatory responses may be important in uncovering the role microglia play in the progression of ASD.

Tanycytes and schizophrenia

The third ventricle provides an excellent route for tanycytes to access all areas of the hypothalamus. The hypothalamus, specifically the ARH, is a key location for retinoic acid (RA) signaling [50-53]. RA is an active oxidized form of vitamin A and participates in embryonic organ growth, cell growth, and cell differentiation. In SCZ, RA may regulate genes responsible for disease progression, notably RA induced 1. Studies have found the absence of cells within the ARH that express the RA synthetic enzymes RALDH1 and 2. However, tanycytes were observed projecting into the ARH; this structure did express RALDH1 and 2 (Table 1). For this reason, it has been proposed that these enzymes are involved in neurogenesis [50,54-56].

**Table 1:** The proposed functions of tanycytes in different locations in the third ventricle of the hypothalamus ARH, arcuate nucleus of the hypothalamus; BBB, blood-brain barrier; BCSFB, blood-cerebrospinal fluid barrier; DMH, dorsomedial nucleus of the hypothalamus; GK, glucokinase; GLUT2, glucose transporter 2; MCT, monocarboxylate transporter; ME, median eminence of the hypothalamus; RA, retinoic acid; RALDH, retinaldehyde dehydrogenase; Tas1r, Taste 1 receptor; VMH, ventromedial nucleus of the hypothalamus.

Location of tanycytes	Proposed functions (with citations)
DMH	Proposed participation in the BBB regulation [6,7,28,30,55]
VMH	Intracellular Ca²⁺ signaling/glucose-responsive Ca²⁺ changes [35-37]; expression of GLUT2 and GK [3,36] (indirect support only; no direct GLUT2/GK confirmation in list); putative receptor-mediated glucosensing via activation of Tas1r2 and Tas1r3 [36]; proposed participation in the BBB regulation [6,7,28,30,55]; possible expression of interleukin-6R/cytokine signaling involvement [23,24,27]
ARH	Proposed participation in the BBB regulation [6,7,28,30,55]; proposed participation in the BCSFB regulation [7,36,38]; RA signaling [50-54]; expression of RA synthetic enzymes RALDH1 and 2 [53,54] (indirect only; no direct RALDH1/2 evidence cited); expression of MCTs for lactate transport [37]; uptake/transport/access of leptin and ghrelin to/from surrounding tissue [28-34]
ME	Proposed participation in the BCSFB regulation [7,38]; uptake/transport/access of leptin for transport to/from ARH [4,28,30,33]

Another gene related to SCZ is *NKX2-1*, which is responsible for making the homeobox protein. This protein is a transcription factor that drives differentiation of cell types during embryonic development, much like RA, and is highly expressed in tanycytes throughout the lifespan of mice [57,58]. *NKX2-1* interacts with many SCZ-associated genes; genes associated specifically with the hypothalamus are clock circadian regulator (*CLOCK*) [59-63], V-erb-b2 avian erythroblastic leukemia viral oncogene homolog 2 (*ERBB2*) [64,65], V-erb-b2 avian erythroblastic leukemia viral oncogene homolog 4 (*ERBB4*) [65,66], and proenkephalin [48,64].

The hallmark social cognitive deficits in people with SCZ are believed to come from overconnectivity in the thalamus, which starts in childhood but generally does not appear until adolescence (Figure 4). This hyperactivation seems to be similar to that in ASD because of immature growth in the frontotemporal pathway [49]. Further research is needed to fully elucidate these pathways for earlier diagnosis and treatment of SCZ.

Neuroendocrine and glucose regulation by tanycytes that may affect brain development and function

**Figure 5:**
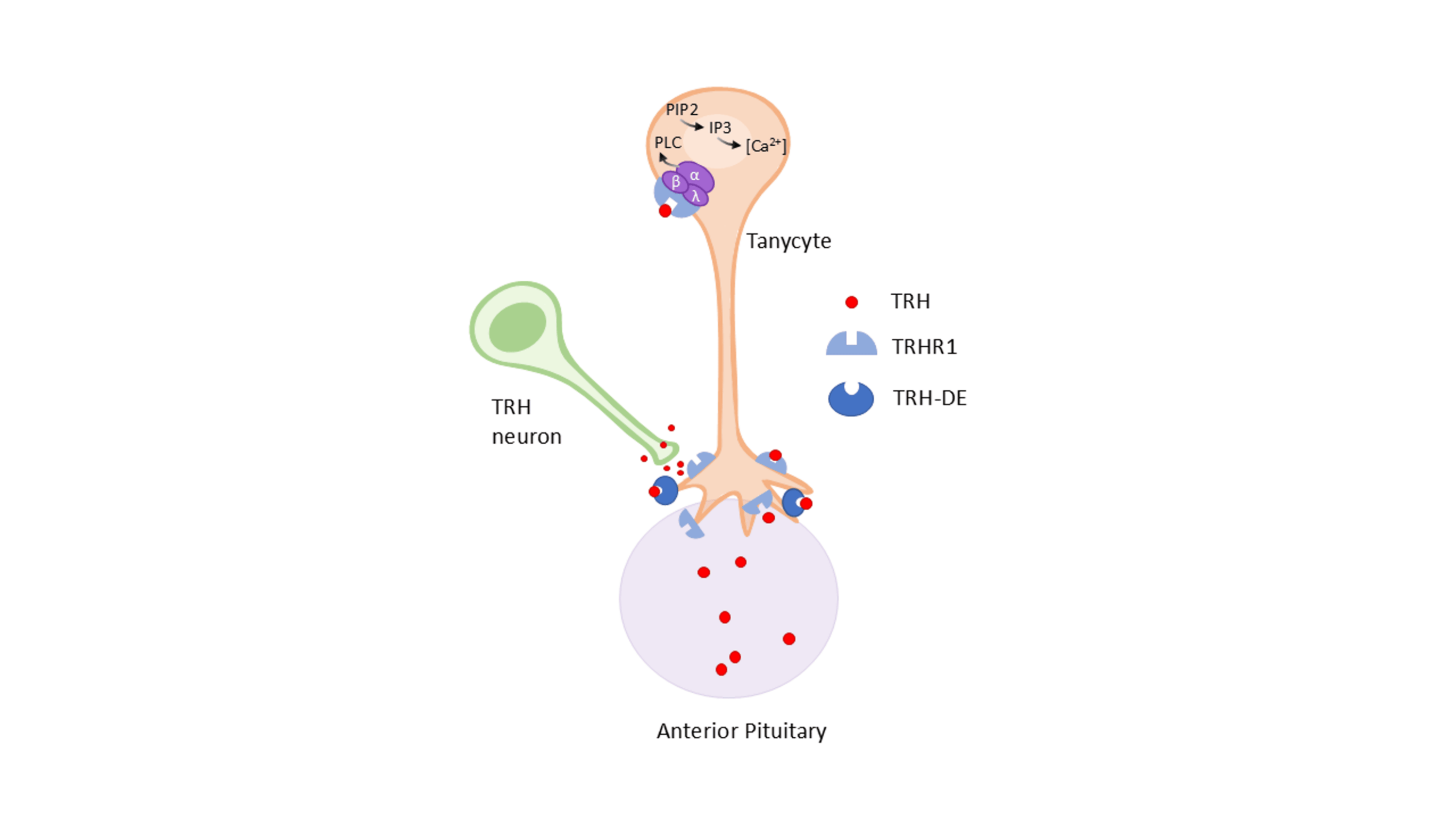
Simplified not-to-scale conceptual schematic diagram of proposed model of hormone regulation by tanycytes in the hypothalamic-pituitary-thyroid axis TRH, thyrotropin-releasing hormone; TRHR1, TRH receptor 1; TRH-DE, TRH-degrading ectoenzyme. Figure/image credit: Leah Grady, Olivia Kelly, and Prasad Dalvi. The figure is prepared using PowerPoint. No AI-assisted tool was used for creating the image.

Tanycytes have been shown to control hormone secretion within the hypothalamic-pituitary-thyroid (HPT) axis (Figure 5), which is responsible for physiological processes, including brain development, cardiovascular function, energy metabolism, and thermoregulation. Situated within the BBB of the ME, tanycytes act as gatekeepers, controlling the crossing of blood-borne substances into the periphery of the brain. They then proceed to function as chemosensors; as chemosensors, tanycytes send alpha- and beta-type processes into the parenchyma [27], thereby influencing neuroendocrine functions. However, more research is necessary to discover the precise mechanisms by which tanycytes regulate the HPT axis in vivo.

Recent advancements in Ca^2+^ imaging have made the study of glucose and its relationship to tanycyte function possible. Tanycytes are believed to have an influence on glucose sensing. The pancreatic beta-cell paradigm is typically responsible for sensing glucose in the body; however, tanycytes also contain all the necessary components to carry out this function. Ca^2+^ imaging studies have demonstrated that tanycytes have a weak reaction to glucose via the beta-cell paradigm. However, more significant findings have emerged from the selective application of glucose onto the cell bodies of tanycytes. This treatment of tanycyte cells resulted in a surge of Ca^2+^ waves with released adenosine triphosphate. This rapid response to various neurotransmitters and ions suggests that tanycytes may serve as biosensors, capable of detecting and responding to changes in their microenvironment [27]. However, it remains unknown whether tanycytes respond to glucose concentration in the CSF or the parenchyma. Knowing how the tanycytes respond - that is, whether they are responsive to glucose concentration in the CSF or in the parenchyma - is crucial for understanding their full significance.

## Conclusions

Advances in neurobiology research have shown that glial cells are a necessary part of the functional components and capabilities of the nervous system. Specifically, the study of tanycytes may be critical for understanding the impact on hypothalamic morphology. Tanycytes may affect metabolic processes and contribute to the etiology of major neurological disorders, which impact social and individual behavior. More mechanistic studies are required, as currently available data are just hypothesis-generating and not mechanistically established.

As discussed, tanycytes could hold the key to understanding various neurological disorders. Going forward, much research is needed to unveil the significance tanycytes have on the HPT axis in vivo and in adult neural stem cells. Additionally, investigating the link between tanycytes and social development disorders may reveal earlier indicators of these disorders. A comprehensive understanding of tanycytes’ involvement in hormone regulation, social behavior, and individual behavior will inform medical practices and treatments in the future.
